# What Is the Effect of Medicaid Expansions on Preexposure Prophylaxis for HIV Prevention Use among Women?

**DOI:** 10.1007/s10461-025-04828-2

**Published:** 2025-07-28

**Authors:** Dion C. Allen, Silvia E. Rabionet, Sannisha K. Dale, Ioana Popovici

**Affiliations:** 1 Department of Sociobehavioral and Administrative Pharmacy, Nova Southeastern University, Fort Lauderdale, FL USA; 2 Culturally-focused HIV Advancements through the Next Generation for Equity (CHANGE) Training Program, University of Miami, Miami, FL USA; 3 Department of Psychology, University of Miami, Coral Gables, FL USA; 4 Department of Public Health Sciences, University of Miami Miller School of Medicine, Miami, FL USA

**Keywords:** Pre-exposure prophylaxis, PrEP, Women, Medicaid, PrEP-to-need ratio, HIV prevention

## Abstract

Pre-exposure prophylaxis (PrEP) is a key biomedical tool for combatting HIV spread, but women account for only 5% of users. Between 2015 and 2019, men saw a 9% decrease in new HIV diagnoses, while rates among women remained constant. Using a difference-in-differences regression model and state level HIV surveillance data (utilizing sex assigned at birth) reported by AIDSVu from 2012 to 2021, we examined the impact of Medicaid expansions under the Affordable Care Act on PrEP outcomes. The study revealed that Medicaid expansions were associated with better PrEP outcomes overall, with increases of 18.9% in PrEP rate and 40.4% in PrEP-to-need ratio for every state that expanded Medicaid. Males experienced better outcomes with a 43% increase in PrEP-to-need ratio, while females saw a 15.6% increase that was not statistically significant. Medicaid expansion improves PrEP access for all, however more targeted strategies are needed to increase PrEP uptake among women vulnerable to HIV.

## Resumen

Profilaxis pre-exposición (PrEP) es crucial para combatir el VIH. Las mujeres representan sólo el 5% de los usuarios. Entre 2015 y 2019, los hombres experimentaron una disminución del 9% en nuevos diagnósticos, no así las mujeres. Utilizando regresión de diferencias en diferencias con datos de AIDSVu entre 2012 y 2021, y datos de vigilancia a nivel estatal, examinamos el impacto de expansiones de *Medicaid*, *Affordable Care Act*, en el uso de PrEP. Las expansiones se asocian con aumentos del 18.9% en el uso y del 40.4% en la tasa relativa de necesidad de PrEP (*PrEP-to-need ratio*) en los estados que expandieron *Medicaid*. Los hombres experimentaron mejores resultados, un aumento del 43% en la tasa relativa de necesidad; las mujeres experimentaron un aumento no significativo del 15.6%. Las expansiones mejoran el acceso a PrEP, sin embargo, se necesitan estrategias para aumentar la aceptación de PrEP entre las mujeres vulnerables al VIH.

## Introduction

In 2019, the US president and administration announced plans to end the HIV epidemic by 2030, aiming for a 90% reduction in new HIV diagnoses across the nation [1, 2]. Preexposure prophylaxis (PrEP) drugs, approved by the FDA in 2012, are used for HIV prevention. PrEP is up to 99% effective against contracting HIV through sexual intercourse and 74% effective against contracting the virus through needle sharing [3–6]. HIV transmission is particularly high among individuals who fall within the low-income bracket, with rates of new HIV diagnoses being especially high among Black individuals [7, 8]. Fortunately, PrEP is covered under Medicaid, making the drug more accessible for those who need it most. The Affordable Care Act (ACA) was signed into law in 2010 and its implementation, including Medicaid expansion, began in 2014 in most states. The program has been helpful in making healthcare accessible, particularly to individuals with low incomes. The ACA expanded eligibility for Medicaid [9] and has provided an opportunity for states to expand coverage to include all adults with income up to 138% of the Federal Poverty Level.

PrEP uptake has been slow since its approval. Of the estimated 1.2 million persons in the U.S with indications for PrEP, only about 18% have been receiving the medication [10]. This issue of low PrEP uptake is even further compounded by the disparity in its use [11], where current data shows that, despite the need, women account for only 5% of PrEP users each year [12–14]. Between 2015 and 2019, we saw a 9% reduction in the annual HIV diagnoses among men, while rates remained stable among women; and a 7% increase in the number of new HIV diagnoses among women who inject drugs, while the rates remained stable among men who inject drugs [11, 15]. These trends indicate an unaddressed vulnerability for HIV among women, whether through sexual transmission or injection drug use, and suggest that women may not be benefitting from PrEP as a preventative measure against HIV.

The relationship between PrEP uptake and affordability for the medication has been documented, with prior studies showing that insured individuals are four times more likely to use PrEP than those uninsured [16]. Evidence also shows that PrEP use is higher in states that have expanded their Medicaid programs [17–19]. However, disparities in PrEP use still exist, even within the population of those with access to insurance through Medicaid. Researchers found racial and gender disparities in PrEP use among Medicaid enrollees in the state of California, with PrEP uptake being highest among White and male individuals [20]. There is inequality in accessing PrEP, even among those who qualify for coverage through Medicaid, and the effect of Medicaid on PrEP use can only be deemed a success if it is expanding access to populations that need it. Women, and especially Black women and those with low incomes, demonstrate a high need for PrEP, but their usage has been low [21, 22].

Existing studies give some indication of the impact of Medicaid expansion on PrEP use. However, most existing research does not account for PrEP use relative to the need for the drug [17, 20]. There are some studies that have taken the need for PrEP into account, but they are limited by the number of years examined in the analysis [18, 19]. Another shortcoming is that PrEP use has not been examined across all states and has therefore been limited to certain geographic areas [20]. Overall, the literature shows that there is a dearth of studies that specifically look at the relationship between Medicaid expansion and PrEP uptake among women using epidemiological data.

Medicaid expansion leads to an increase in insurance coverage for PrEP [23, 24]. This lowers the total cost of PrEP by reducing both the direct cost of the medication, as well as the cost of obtaining a prescription, making PrEP more affordable for disadvantaged populations. In light of the gender disparity in PrEP uptake [11], we sought to determine the effect of Medicaid expansion on PrEP outcomes for cisgender men versus cisgender women. We hypothesized that women that are vulnerable to HIV and who are living in states that expanded Medicaid programs are more likely to seek PrEP treatment (*proxied by PrEP rate and PrEP-to-Need ratio*) due to increased access to treatment and treatment affordability.

## Methods

### Datasets and Variables

#### Outcome Variables

The study was conducted using publicly available deidentified data across multiple sources that were matched and merged at the state/year level. We used aggregate state-level epidemiological data from 2012, the year when PrEP was approved for HIV prevention, through 2021. The two outcome measures were the state/year PrEP rate and PrEP-to-need ratio (PNR), both obtained from the AIDSVu website [25, 26]. HIV surveillance data is reported by AIDSVu and is managed by the Center for AIDS Research at Emory University (CFAR) and the Rollins School of Public Health with Gilead Sciences Inc. A total of 54,000 pharmacies, 1,500 hospitals, 800 outpatient clinics, and 80,000 doctor’s offices nationwide provide data on PrEP use.

PrEP rate data indicates the number of PrEP users aged 13 and older, per 100,000 state population, who were prescribed PrEP medication. The three PrEP rate categories that we considered were the general state population (a combination of men and women), men only, and women only, as reported by AIDSVu [25, 26]. The data defines sex as sex assigned at birth. We also examined the PNR because PrEP rate data is a basic indicator of PrEP use and does not suggest the distribution of PrEP in relation to epidemic need. The PNR indicates the level of PrEP use relative to the need for the drug. It is calculated by dividing the number of PrEP users in a given state each year by the number of new HIV diagnoses in the state for that year [25, 26]. Greater PrEP coverage is indicated by a higher PNR. Like the PrEP rate, PNR is calculated for all Americans 13 years of age and older and is broken down by geography and sex at the state level.

1 $$\:PrEP\:rate=\left(\frac{\text{N}\text{o}.\:\text{o}\text{f}\:\text{P}\text{r}\text{E}\text{P}\:\text{U}\text{s}\text{e}\text{r}\text{s}}{\text{P}\text{o}\text{p}\text{u}\text{l}\text{a}\text{t}\text{i}\text{o}\text{n}\:13\:\text{a}\text{n}\text{d}\:\text{a}\text{b}\text{o}\text{v}\text{e}}\right)\times\:100000$$

2 $$\:PNR=\frac{\text{N}\text{o}.\:\text{o}\text{f}\:\text{P}\text{r}\text{E}\text{P}\:\text{U}\text{s}\text{e}\text{r}\text{s}}{\text{N}\text{o}.\:\text{o}\text{f}\:\text{n}\text{e}\text{w}\:\text{H}\text{I}\text{V}\:\text{d}\text{i}\text{a}\text{g}\text{n}\text{o}\text{s}\text{e}\text{s}}$$

### Key Independent Variable

The main predictor was any state ACA-Medicaid expansion that occurred between 2012 and 2021. The Kaiser Family Foundation provides historical data on each state’s choice to expand [27]. Forty states and the District of Colombia have ratified the expansion as of May 9, 2025. Table 1 lists each state and the dates of expansion that occurred within this study’s analysis period. Although most expansions took place on January 1st, 2014, other states adopted later [28–30]. Some states (e.g., Maine) adopted the expansion with retroactive coverage. Other states (e.g., South Dakota or South Carolina) implemented coverage more recently, outside our analysis period. For this study, Medicaid expansion states were defined as states that adopted Medicaid under the ACA during the analysis period (2012–2021). Additionally, retroactive coverage was excluded with the assumption that it would have no effect on PrEP outcomes.

**Table 1:** ACA medicaid expansion States effective dates

State	Medicaid expansion date
Alaska	9/1/2015
Arizona, Arkansas, California, Colorado, Connecticut, Delaware, District of Columbia, Hawaii, Illinois, Iowa, Kentucky, Maryland, Massachusetts, Minnesota, Nevada, New Jersey, New Mexico, New York, North Dakota, Ohio, Oregon, Rhode Island, Vermont, Washington, West Virginia	1/1/2014
Idaho, Utah	1/1/2020
Indiana	2/1/2015
Louisiana	7/1/2016
Maine	1/10/2019
Michigan	4/1/2014
Missouri	10/1/2021
Montana	1/1/2016
Nebraska	10/1/2020
New Hampshire	8/15/2014
Oklahoma	7/1/2021
Pennsylvania	1/1/2015
Virginia	1/1/2019
Expansions adopted outside of the analysis period	
North Carolina	12/1/2023
South Dakota	7/1/2023

Notes: As of May 9, 2025, 40 states and the District of Colombia have expanded Medicaid under the ACA and the remaining 10 have not (Alabama, Florida, Georgia, Kansas, Mississippi, South Carolina, Tennessee, Texas, Wisconsin, Wyoming). North Carolina (December 1, 2023) and South Dakota (July 1, 2023) expanded in 2023 but were outside of the study analysis period. Source: Kaiser Family Foundation (Accessed June 10, 2025 https://www.kff.org/health-reform/state-indicator/state-activity-around-expanding-medicaid-under-the-affordable-care-act/?currentTimeframe=0&sortModel=%7B%22colId%22:%22Location%22,%22sort%22:%22asc%22%7D)

Another assumption was that the ACA Medicaid expansion would not have an immediate effect after adoption. We reasoned that it would take some time for those gaining access to Medicaid insurance to actually obtain insurance coverage and to begin using PrEP. We created a dichotomous variable for Medicaid expansions, assigning a value of zero for each year prior to expansion and one in the Medicaid adoption year and all years post-adoption. Based on the assumption of a time delay between expansion and PrEP uptake, the expansion variable was lagged by one year.

### Control variables

#### Access To Healthcare Services

Regressions included the number of mental health and substance use disorder (SUD) treatment facilities per 1000 state HIV cases ([Number of mental health and SUD treatment centers/number of HIV cases in the state] x1000) [31, 32] and the number of family planning clinics per 100,000 state population ([number of family planning clinics/state population] x100000) [31, 33] as proxies for healthcare services. The North American Industry Classification System (NAICS) was used to identify the number of facilities on County Business Patterns (CBP) by industry code (outpatient mental health and SUD centers [code 62142]); family planning clinics [code 62141]) (U.S. Census Bureau, 2021). The data was available from 2012 to 2020. Year 2021 data was imputed using year 2020 data. The state HIV statistics were used to estimate the availability of mental health and SUD treatment facilities, including all HIV cases, HIV cases among males, and HIV cases among females [32]. Similarly, all individuals 13 years of age and older, males 13 years of age and older, and females 13 years of age and older made up the state population data used to estimate the availability of family planning clinics [33].

#### Demographic Controls

We also controlled for state-level demographics including age, race, ethnicity, income, marital status and education. The age variable consisted of all individuals aged 13 years and above. This was done to match the data with the outcome variables, which included those aged 13 years and above who were prescribed PrEP for HIV prevention. Race was divided among individuals who identified as White, African American or Other race (all non-White and non-African American races). Ethnicity was a dichotomous variable indicating individuals’ ethnic origin (Hispanic or non-Hispanic origin). Income referred to participants’ annual household income. Marital status was categorized into individuals who were single/never married, married or divorced. Education consisted of individuals with less than high school education, high school graduates, those with some college education or college graduates. The individual-level demographics were collected from the Current Population Survey (CPS), Integrated Public Use Microdata Series (IPUMS) [34], a monthly survey that is administered to community-dwelling U.S. residents. The data was then aggregated at the state level.

### Empirical Model

A two-way fixed-effects (TWFE) regression model was used to model the effect of Medicaid expansion on PrEP rate and PNR as outlined in Eqs. (3) and (4):

3 $$\begin{aligned} \:{\text{PrEP}}\:{\text{Rate}}_{{{\text{st}}}} &= \alpha _{0} + {\text{Expand}}_{{{\text{st}}}} \alpha _{1} + {\text{X}}_{{{\text{st}}}} \alpha _{{\text{x}}} \\ & + \theta _{{\text{s}}} + \tau _{{\text{t}}} + \mu _{{{\text{s}},{\text{t}}}} \\ \end{aligned} $$

4 $$\begin{aligned} \:{\text{PNR}}_{{{\text{st}}}} &= \alpha _{0} + {\text{Expand}}_{{{\text{st}}}} \alpha _{1} \\ & + {\text{X}}_{{{\text{st}}}} \alpha _{{\text{x}}} + \theta _{{\text{s}}} + \tau _{{\text{t}}} + \mu _{{{\text{s}},{\text{t}}}} \\ \end{aligned} $$

The PrEP rate for the study population (all, men, women) in state *s* in year *t* was denoted by $\:{PrEP\:Rate}_{st}$. The PNR for the study population in state *s* in year *t* was denoted by $\:{PNR}_{st}$. We used estimates for the entire population, males only, and females only. The primary explanatory variable, $\:{Expand}_{st}$, was a measure of whether state *s* expanded Medicaid in year *t*. To account for the time lag between the implementation of Medicaid expansion and its impact on the PrEP rate and PNR through PrEP uptake, we lagged the variable by one year. This was done under the presumption that the program’s effects would plausibly affect the outcomes in the year following the expansion year. State characteristics (age, race, ethnicity, income, marital status, and education) and the availability of family planning clinics and mental health SUD treatment centers at the state level were among the control variables represented by the vector $\:{X}_{st}$. $\:{\theta\:}_{s}$ was a vector of state fixed effects and $\:{\tau\:}_{t}$ were year fixed effects. State fixed effects accounted for time-invariant unobservable state-level variables that might capture state sentiment and attitudes toward HIV. National secular trends in PrEP use were captured by year fixed effects.

#### Event-study Model

An assumption for difference-in-differences estimators to provide causal estimates is that if the treatment group had not received treatment, the control and treatment groups would have trended similarly. In other words, the control group can offer the treatment group a counterfactual trend following Medicaid expansion (‘parallel trends’). We provided suggestive evidence on the ability of the data to meet the parallel trends assumption by estimating event-study models.

The event-study model equations were as follows:

5 $$ \begin{aligned} \:PrEP\:Rate_{{st}} &= \beta _{0} + \sum {_{{j = 1}}^{{12}} } \delta _{j} Rel\_time\_expansion_{{s,j}} \\ & + X_{{st}} \beta _{x} + \theta _{s} + \tau _{t} + \varepsilon _{{st}} \\ \end{aligned} $$

6 $$ \begin{aligned} PNR_{{st}} &= \beta _{0} + \sum {_{{j = 1}}^{{12}} } \delta _{j} Rel\_time\_expansion_{{s,j}} \\ & + X_{{st}} \beta _{x} + \theta _{s} + \tau _{t} + \varepsilon _{{st}} \\ \end{aligned} $$

We centered the data around the event (i.e., Medicaid expansion) for states that adopted and implemented Medicaid. We defined the year of the event (e) as the first full year that the Medicaid expansion was in effect. We created single-year bins corresponding to six or more years pre-expansion through six or more years post-expansion. We thus included six policy leads and six policy lags in our event-study. States that did not expand Medicaid were coded as zero for all lead and lag years. The one-year pre-expansion lead was the excluded/comparison group. The focus was on coefficient estimates for lags, which revealed post-expansion effects or differential post-trends.

## Results

Due to the dataset’s classification of sex, all sex-specific analyses presented below should be interpreted as referring to cisgender individuals. Table 2 reports the average values for all variables across three categories: all states, expansion states (adopted and implemented ACA Medicaid expansion within the analysis period, 2012–2021) and non-expansion states (did not expand during analysis period). The unit of observation is a state in a year (state/year).

**Table 2:** Summary statistics of state/year level averages for all variables, 2012–2021

	All States	Expansion States	Nonexpansion States	t-test
PrEP rate – State**	55.2	58.3	48.4	2.522
PrEP rate – Male**	96.7	102.2	84.3	2.287
PrEP rate – Female*	8.20	7.81	9.06	1.687
PNR – State***	4.45	5.14	2.92	3.814
PNR – Male***	4.89	5.66	3.19	3.705
PNR – Female**	2.04	2.31	1.42	2.805
ACA Medicaid Expansion	0.50	0.72	0	--
FP clinics	0.88	0.92	0.78	4.082
MHSUD treatment	17.3	19.6	12.3	− 0.574
Male*	0.48	0.49	0.48	1.770
Female*	0.52	0.51	0.52	-1.770
Age*	36.6	36.6	36.8	1.686
White	0.77	0.78	0.76	1.026
African American***	0.13	0.11	0.18	-5.921
Other Race***	0.10	0.12	0.065	4.602
Hispanic**	24.3	23.3	26.5	2.444
Income***	8.88	9.29	7.96	6.881
Married	0.41	0.40	0.41	-1.062
Divorced***	0.13	0.13	0.14	-4.081
Never Married***	0.46	0.47	0.45	3.547
Less than high school***	0.18	0.17	0.19	-5.118
High school graduate**	0.27	0.27	0.28	-2.297
Some college***	0.26	0.26	0.27	-5.253
College graduate***	0.29	0.30	0.26	6.822
**Observations**	**510**	**380**	**130**	-

Notes: The unit of observation is a state in a year (PrEP rate-State, PrEP rate-Male, PrEP rate-Female = average PrEP rate for all states, all males, all females, respectively) (PNR-State, PNR-Male, PNR-Female = average PNR for all states, all males, all females, respectively). ACA Medicaid Expansion is coded one for states that expanded Medicaid in a state in a year and zero for states that did not. Expansion refers to states that have adopted and implemented ACA Medicaid expansion by December 31, 2021. Non-expansion refers to states that did not adopt ACA Medicaid expansion by the same date. MHSUD treatment = (Number of mental health and substance use disorder treatment centers/number of HIV cases in the state) x1000. FP clinics = (number of family planning clinics/state population) x100000. Income = annual income divided by 10,000. t-test was used for differences between group means. ***, **, and * = statistically different from zero at the 1%, 5%, and 10% level

The study found that women constituted 51% and 52% of expansion and nonexpansion states (t-test= -1.770, *p* < 0.10), respectively. The average age was similar across states (expansion states = 36.6 and nonexpansion states = 36.8, t-test = 1.686, *p* < 0.10). Nonexpansion states had a higher proportion of African Americans (0.18, t-test= -5.921, *p* < 0.01) and Hispanic ethnicity (26.5, t-test = 2.444, *p* < 0.05), while expansion states had a higher representation of Other race individuals (0.12, t-test = 4.602, *p* < 0.01). Expansion states had a higher average annual household income, with an average of $92,900, compared to nonexpansion states $79,600 (t-test = 6.881, *p* < 0.01). Nonexpansion states had a higher proportion of divorced individuals (0.14, t-test= -4.081, *p* < 0.01), while expansion states had a higher proportion of those who were never married (0.47, t-test = 3.547, *p* < 0.01). Additionally, expansion states had a higher percentage of college graduates (0.30, t-test = 6.822, *p* < 0.01) compared to nonexpansion states.

The average PrEP outcomes (PrEP rate and PNR) are reported for males, females and for the general state population. The majority of the observations (*n* = 510) were represented by states that expanded Medicaid (*n* = 380). The average yearly PrEP rate for the general population was 55.2. We observed a higher average PrEP rate among states that expanded (58.3) compared with states that did not expand (48.4) (t-test = 2.522, *p* < 0.05). Additionally, the PrEP rate was greater among males in all three groups: all states (males = 96.7, females = 8.20), states that expanded Medicaid (males = 102.2, t-test = 2.287, *p* < 0.05; females = 7.81, t-test = 1.687, *p* < 0.10) and states that did not expand (males = 84.3, t-test = 2.287, *p* < 0.05; females = 9.06, t-test = 1.687, *p* < 0.10). For both sexes, the average yearly PNR was 4.45. Medicaid expansion states had a higher average PNR (5.14), compared with states that did not expand (2.92) (t-test = 3.814, *p* < 0.01). Males consistently had a higher PNR (4.89; 5.66; 3.19, t-test = 3.705, *p* < 0.01) than females (2.04; 2.31; 1.42, t-test = 2.805, *p* < 0.05) across all states, in states that expanded Medicaid and in states that did not expand.

### Difference-in-Differences (DD) Regression Models

The difference-in-differences (DD) regression results for the effect of Medicaid expansion on PrEP rate are reported in Table 3. PrEP rate increased across all groups (state, males and females) post-expansion. The population as a whole, males, and females had average PrEP rates of 55.2, 96.7, and 8.2, respectively. All things considered, expansion was associated with a 10.458 (18.9%, t-test = 4.84, *p* < 0.01) increase in the PrEP rate for the general population, 17.073 (17.7%, t-test = 4.31, *p* < 0.01) for males and 0.621 (8%, t-test = 2.36, *p* < 0.05) for females.

**Table 3:** Effect of state ACA-Medicaid expansions on PrEP rate using a DD model, 2012–2021

	State	t-test	Male	t-test	Female	t-test
	PrEP Rate		PrEP Rate		PrEP Rate	
Medicaid Expansion	10.458***	4.84	17.073***	4.31	0.621**	2.36
	[6.209,14.708]		[9.280,24.865]		[0.104,1.139]	
Mean expansion state	55.2		96.7		8.20	
Percentage change	18.9%		17.7%		8%	
Controls						
FP clinics^a^	-2.341	-0.58	-5.695	-0.77	-1.578***	-3.23
	[-10.233,5.550]		[-20.166,8.776]		[-2.539,-0.617]	
MHSUD treatment^b^	0.529***	5.30	1.036***	5.66	0.033***	2.72
	[0.333,0.726]		[0.676,1.396]		[0.009,0.057]	
Age	1.976	1.45	5.946**	2.38	0.252	1.52
	[-0.701,4.653]		[1.036,10.855]		[-0.074,0.578]	
African American	40.510	0.91	33.587	0.41	-1.805	-0.33
	[-47.449,128.468]		[-127.709,194.883]		[-12.521,8.911]	
Other Race	153.870***	3.33	269.660***	3.18	17.410***	3.09
	[62.929,244.811]		[102.896,436.424]		[6.331,28.489]	
Hispanic	-0.176	-1.56	-0.300	-1.45	-0.018	-1.30
	[-0.397,0.046]		[-0.707,0.107]		[-0.045,0.009]	
Income	3.667***	2.73	6.555***	2.66	0.297*	1.82
	[1.028,6.307]		[1.715,11.395]		[-0.024,0.619]	
Married	-156.579*	-1.86	-414.153***	-2.68	-16.573	-1.62
	[-321.928,8.771]		[-717.366,-110.941]		[-36.717,3.571]	
Divorced	-200.066*	-1.77	-545.408***	-2.63	-38.013***	-2.75
	[-422.735,22.602]		[-953.729,-137.087]		[-65.140,-10.886]	
High school graduate	18.965	0.29	-6.865	-0.06	6.912	0.88
	[-107.860,145.790]		[-239.433,225.702]		[-8.539,22.363]	
Some college	-76.693	-1.24	-126.056	-1.12	4.376	0.58
	[-197.763,44.378]		[-348.071,95.958]		[-10.374,19.126]	
College graduate	60.909	0.96	119.896	1.04	13.084*	1.70
	[-63.167,184.985]		[-107.630,347.422]		[-2.032,28.200]	
**Observations**	**510**		**510**		**510**	

Notes: The unit of observation is a state in a year. Medicaid Expansion is coded one for states that expanded Medicaid in a state in a year and zero for states that did not. All models estimated with OLS and control for state demographics, state and year fixed effects. 95% confidence intervals are reported in parentheses. ***, **, and * = statistically different from zero at the 1%, 5%, and 10% level

^a^FP = Family planning

^b^MHSUD = Mental health and substance use disorder

Several control variables also showed statistical significance. Increased availability of family planning clinics was associated with lower PrEP rates among females, while increased availability of mental health and SUD treatment centers was associated with increased PrEP rate across state, males and females. States with a larger proportion of individuals of other race and/or with higher average incomes had higher PrEP rates. Also, states with lower proportions of married or divorced individuals showed lower PrEP rates. Within the male population, states that had a higher proportion of older aged males, males of other race and/or those with higher incomes showed higher PrEP rates. Conversely, states with higher proportions of married or divorced males showed lower PrEP rates. Lastly, we found higher PrEP rates among states with a higher proportion of other race females, those of higher income and/or those who were college graduates, but lower PrEP rates among states with a higher proportion of divorced females.

The findings of the DD regression for the impact of Medicaid expansion on PNR are displayed in Table 4. The data was weighted by the state population of those aged 13 and above. We saw an increase in PNR across all groups. For every state that expanded Medicaid, there was a 40% (t-test = 3.37, *p* < 0.01) increase in PNR for the overall population. As with the PrEP rate results, this increase seemed to be mostly due to a rise in PNR for males, who saw a 43% (t-test = 3.17, *p* < 0.01) increase in PNR over the pre-expansion baseline. For females, the expansion was associated with a 15.6% (t-test = 1.67, *p* = 0.106) increase in PNR. Although this PNR estimate for females did not reach statistical significance, the p-value (*p* = 0.106) approached conventional levels of statistical significance, suggesting a potential association. The lack of statistical significance may be due to limited statistical power as the sample size was modest and the model included numerous covariates which can reduce degrees of freedom and increase the data requirements for detecting statistically significant effects.

**Table 4:** Effect of state ACA-Medicaid expansions on PNR using a DD model, 2012–2021

	State	t-test	Male	t-test	Female	t-test
	PNR		PNR		PNR	
Medicaid Expansion	1.798***	3.37	2.104***	3.17	0.318	1.67
	[0.726,2.869]		[0.771,3.437]		[-0.065,0.701]	
Mean expansion state	4.45		4.89		2.04	
Percentage change	40.4%		43%		15.6%	
***Controls***						
FP Clinics^a^	-0.962	-0.43	-1.371	-0.50	-0.062	-0.05
	[-5.416,3.491]		[-6.866,4.123]		[-2.426,2.301]	
MHSUD treatment^b^	-0.118*	-1.93	-0.150*	-1.89	-0.047	-1.47
	[-0.240,0.005]		[-0.309,0.009]		[-0.111,0.017]	
Age	-0.369	-1.10	-0.577	-1.33	0.087	0.49
	[-1.040,0.303]		[-1.446,0.292]		[-0.271,0.444]	
African American	12.335	1.07	16.348	1.14	1.713	0.38
	[-10.739,35.410]		[-12.581,45.277]		[-7.359,10.784]	
Other Race	-4.286	-0.38	-5.674	-0.41	0.023	0.00
	[-27.232,18.661]		[-33.295,21.947]		[-13.337,13.384]	
Hispanic	-0.003	-0.11	-0.004	-0.15	0.017	0.87
	[-0.049,0.044]		[-0.057,0.049]		[-0.022,0.057]	
Income	1.162***	3.12	1.534***	3.16	0.037	0.09
	[0.414,1.910]		[0.560,2.508]		[-0.758,0.832]	
Married	7.782	0.37	13.978	0.54	-2.559	-0.29
	[-34.632,50.196]		[-37.827,65.782]		[-20.486,15.369]	
Divorced	0.511	0.02	4.326	0.14	-12.352	-0.87
	[-50.708,51.730]		[-56.262,64.914]		[-40.985,16.282]	
High school graduate	0.910	0.06	-0.832	-0.04	0.818	0.08
	[-29.575,31.394]		[-39.944,38.280]		[-19.942,21.577]	
Some college	-39.121**	-2.58	-46.025**	-2.67	-7.113	-0.91
	[-69.560,-8.682]		[-80.621,-11.429]		[-22.888,8.663]	
College graduate	-6.808	-0.35	-15.171	-0.61	14.864	1.28
	[-45.624,32.009]		[-64.958,34.616]		[-8.523,38.250]	
**Observations**	**510**		**510**		**508**	

Notes: The unit of observation is a state in a year. Medicaid Expansion is coded one for states that expanded Medicaid in a state in a year and zero for states that did not. All models estimated with OLS and control for state demographics, state and year fixed effects. 95% confidence intervals are reported in parentheses. ***, **, and * = statistically different from zero at the 1%, 5%, and 10% level

^a^FP = Family planning

^b^MHSUD = Mental health and substance use disorder

When the impact of Medicaid expansion on PNR was evaluated, the results also revealed statistical significance for some control variables of the state and male populations. We saw a higher PNR in states with higher average incomes but a lower PNR in states that had a greater availability of mental health and SUD treatment and higher proportions of individuals with some college education. No controls showed statistical significance among females.

The event study model results for Medicaid expansion on the PrEP rate are shown in Table 5. The lack of statistical significance of the leads estimates brought evidence of no pre-treatment trends. The only statistically significant pre-expansion estimates were for four years for the general state population (-8.767, t-test=-1.94, *p* < 0.10) and for the male population (-16.107, t-test=-1.89, *p* < 0.10), which suggested no difference between expansion and non-expansion states prior to expansion. The estimates for the lags, however, revealed statistically significant differences between expansion and non-expansion states; expansion increased PrEP rate for the general population, males and females. For males, the effect of the expansion seemed to increase in magnitude in the years post expansion with increasingly stronger effects as years passed.

**Table 5:** Effect of state ACA-Medicaid expansions on PrEP rate using an event study model, 2012–2021

	State	t-test	Male	t-test	Female	t-test
	PrEP Rate		PrEP Rate		PrEP Rate	
6 + years pre-expansion	-6.149	-1.47	-10.937	-1.38	0.637	1.04
	[-14.371,2.074]		[-26.505,4.632]		[-0.569,1.844]	
5 years pre-expansion	-7.465	-1.43	-11.425	-1.16	0.707	0.93
	[-17.691,2.761]		[-30.786,7.936]		[-0.794,2.208]	
4 years pre-expansion	-8.767*	-1.94	-16.107*	-1.89	0.190	0.29
	[-17.645,0.111]		[-32.879,0.666]		[-1.110,1.490]	
3 years pre-expansion	-4.579	-1.20	-6.396	-0.88	0.088	0.16
	[-12.111,2.952]		[-20.657,7.864]		[-1.017,1.194]	
2 years pre-expansion	-2.786	-0.99	-3.216	-0.61	-0.262	-0.64
	[-8.299,2.727]		[-13.645,7.214]		[-1.071,0.546]	
Expansion year	0.443	0.16	3.170	0.59	0.183	0.44
	[-5.127,6.013]		[-7.375,13.715]		[-0.635,1.000]	
1 year post expansion	0.715	0.24	2.862	0.50	0.078	0.18
	[-5.202,6.632]		[-8.339,14.063]		[-0.790,0.946]	
2 years post expansion	2.467	0.76	10.834*	1.77	0.676	1.42
	[-3.896,8.829]		[-1.217,22.885]		[-0.258,1.610]	
3 years post expansion	8.896***	2.61	24.366***	3.78	0.824	1.65
	[2.199,15.593]		[11.683,37.050]		[-0.159,1.807]	
4 years post expansion	17.290***	5.11	31.899***	4.98	0.668	1.34
	[10.634,23.946]		[19.299,44.499]		[-0.309,1.644]	
5 years post expansion	21.928***	6.30	38.692***	5.87	1.543***	3.02
	[15.087,28.769]		[25.741,51.643]		[0.539,2.546]	
6 + years post expansion	23.912***	6.84	42.875***	6.48	1.208**	2.35
	[17.043,30.781]		[29.866,55.884]		[0.199,2.216]	
**Observations**	**510**		**510**		**510**	

Notes: The unit of observation is a state in a year. All models estimated with OLS and control for state demographics, state and year fixed effects. Confidence intervals that account for within-state clustering are reported in parentheses. ***, **, and * = statistically different from zero at the 1%, 5%, and 10% level

Table 6 reports the event study model for the impact of Medicaid expansion on PNR. The findings indicated that all populations experienced increase in PNR post-expansion, with stronger effects for the male compared to the female population. Finally, similar to the PrEP rate, the effect of the expansion on PNR seemed to increase in magnitude in the years post expansion.

**Table 6:** Effect of state ACA-Medicaid expansions on PNR using an event study model, 2012–2021

	State PNR	t-test	Male PNR	t-test	Female PNR	t-test
6 + years pre-expansion	-0.939	-1.48	-1.691**	-2.35	0.423	1.51
	[-2.188,0.311]		[-3.107,-0.276]		[-0.126,0.973]	
5 years pre-expansion	-0.407	-0.52	-1.231	-1.37	0.663*	1.91
	[-1.961,1.146]		[-2.991,0.529]		[-0.020,1.346]	
4 years pre-expansion	0.011	0.02	-0.423	-0.54	0.296	0.98
	[-1.335,1.356]		[-1.947,1.102]		[-0.295,0.888]	
3 years pre-expansion	0.387	0.67	0.112	0.17	-0.046	-0.18
	[-0.757,1.532]		[-1.184,1.409]		[-0.549,0.456]	
2 years pre-expansion	0.143	0.34	0.275	0.57	0.163	0.87
	[-0.694,0.980]		[-0.674,1.223]		[-0.205,0.531]	
Expansion year	0.157	0.37	0.146	0.30	0.356*	1.88
	[-0.689,1.004]		[-0.813,1.105]		[-0.017,0.729]	
1 year post expansion	0.776*	1.70	0.947*	1.83	0.343*	1.71
	[-0.123,1.675]		[-0.071,1.966]		[-0.052,0.738]	
2 years post expansion	1.144**	2.32	1.426**	2.56	0.334	1.54
	[0.177,2.111]		[0.331,2.522]		[-0.092,0.760]	
3 years post expansion	1.933***	3.73	1.919***	3.27	0.113	0.50
	[0.915,2.950]		[0.766,3.073]		[-0.335,0.560]	
4 years post expansion	2.663***	5.18	3.654***	6.27	0.529**	2.34
	[1.652,3.674]		[2.508,4.799]		[0.084,0.973]	
5 years post expansion	3.027***	5.73	3.775***	6.30	0.685***	2.95
	[1.988,4.066]		[2.598,4.953]		[0.228,1.142]	
6 + years post expansion	1.740***	3.28	2.122***	3.53	0.535**	2.29
	[0.697,2.784]		[0.939,3.305]		[0.075,0.994]	
**Observations**	**510**		**510**		**508**	

Notes: The unit of observation is a state in a year. All models estimated with OLS and control for state demographics, state and year fixed effects. Data are weighted by the state population aged 13 years and older. Confidence intervals that account for within-state clustering are reported in parentheses. ***, **, and * = statistically different from zero at the 1%, 5%, and 10% level

**Fig. 1:**
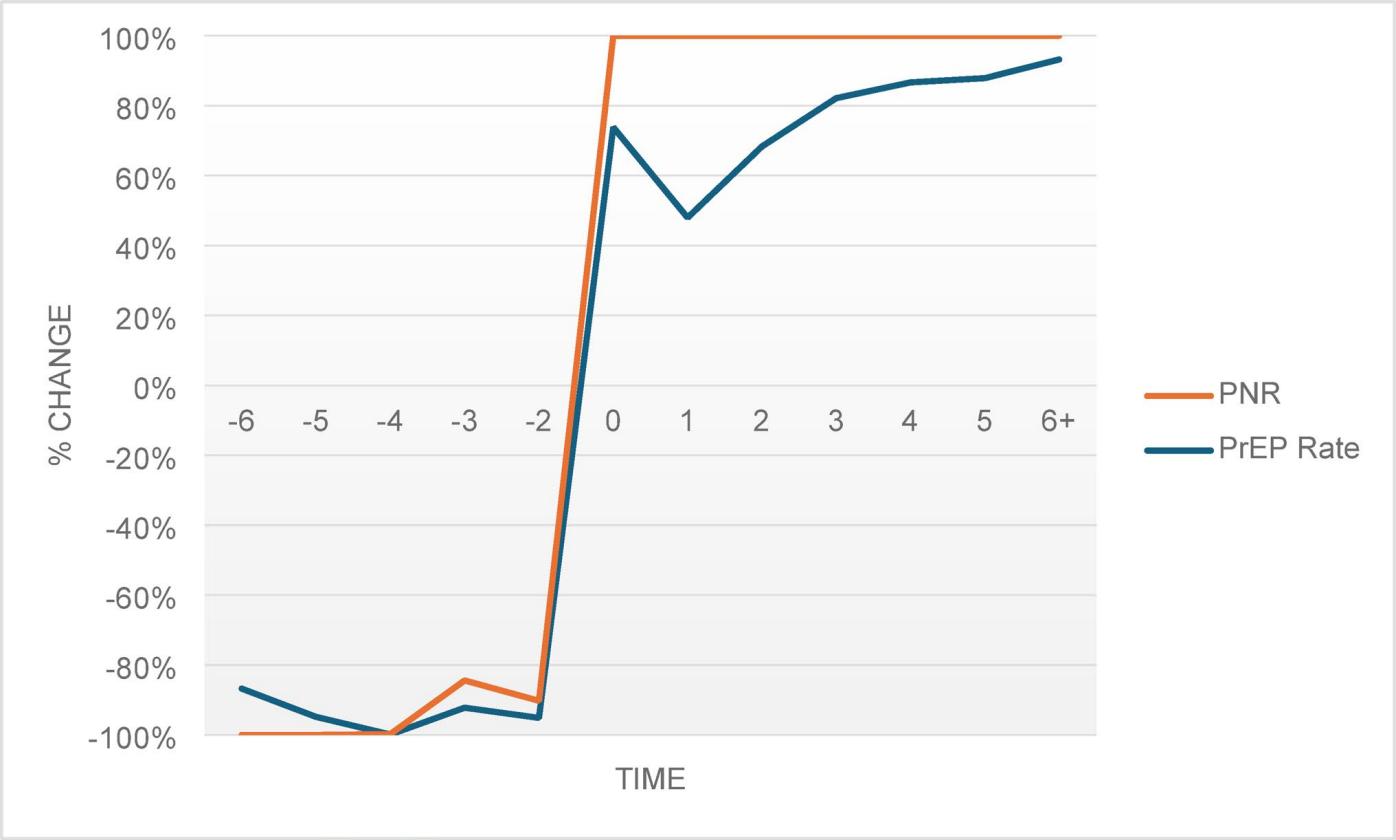
Effect of Medicaid expansion on changes in PrEP rate and PNR – State. Note: Pattern of changes from the event study model shown with − 6 representing six or more years pre-expansion and + 6 representing six or more years post-expansion

**Fig. 2:**
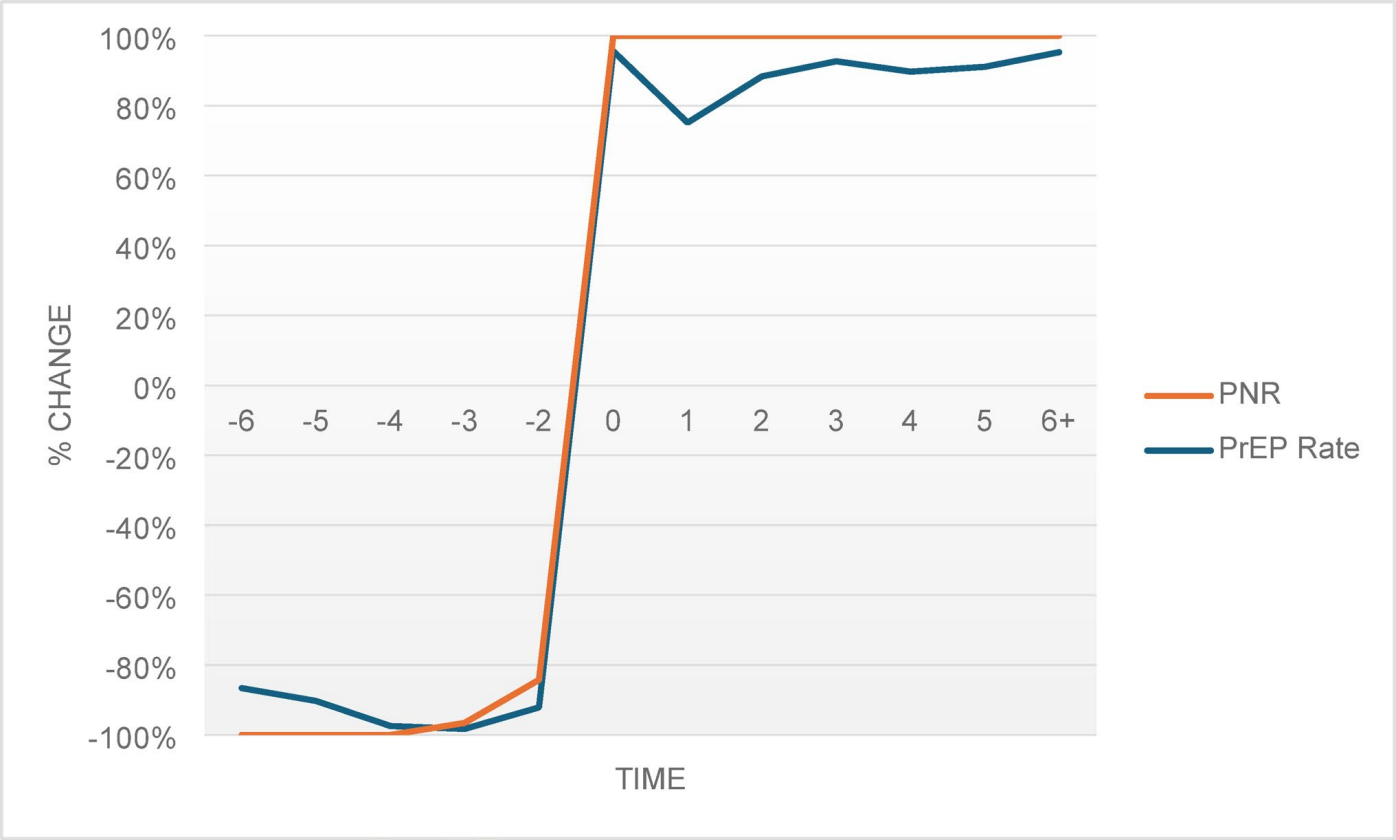
Effect of Medicaid expansion on changes in PrEP rate and PNR – Males. Note: Pattern of changes from the event study model shown with − 6 representing six or more years pre-expansion and + 6 representing six or more years post-expansion

**Fig. 3:**
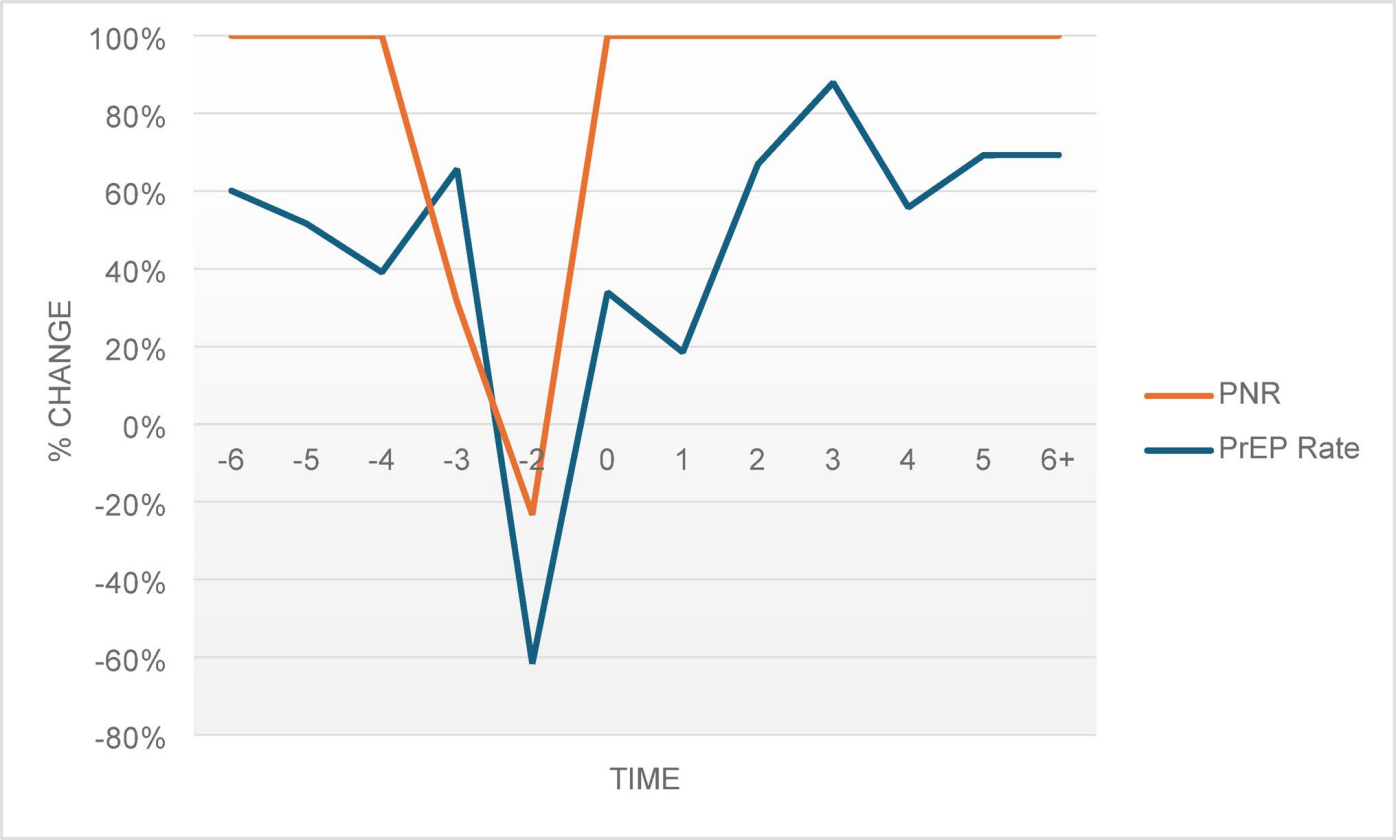
Effect of Medicaid expansion on changes in PrEP rate and PNR – Females. Note: Pattern of changes from the event study model shown with − 6 representing six or more years pre-expansion and + 6 representing six or more years post-expansion

The interpretation of the PNR coefficients is critical as the PNR is a metric calculated by dividing the number of PrEP users by the number of HIV diagnoses in a state. It was unclear whether changes in the outcomes were due to changes in the numerators, denominators, or both. To determine this, comparisons of the results in the event study models were conducted, observing the pattern of direction of changes in PrEP rate and PNR (See Figs. 1 and 2, & 3). All three figures showed similar patterns over time, indicating that changes in PNR were due to PrEP prescriptions, not HIV diagnoses. Figure 3, however, showed less precision for the population of females, but this was not surprising as the resulting statistically significant trend for Medicaid expansion on PNR among females in the event study model were not as strong as the coefficients for males.

## Discussion

This study supports the importance of Medicaid expansion on health outcomes in the U.S [17–19]. It found that Medicaid expansion positively impacted PrEP use (PrEP rate and PNR), increasing PrEP use in general and for both men and women. While this finding is consistent with previous studies demonstrating this association between Medicaid and PrEP use [17, 18, 20, 35, 36], this paper contributes to the existing literature as few of the existing studies account for need-based measures or include all states. Moreover, extant literature is limited by short timeframes, geographic scope, or lack of focus on women using epidemiological data. The main goal of Medicaid expansion is to improve health outcomes through expanded healthcare access. Medicaid expansion offers health insurance to a large population who would otherwise not be able to afford proper healthcare. Cost remains a substantial obstacle for many individuals at risk for HIV, and Medicaid coverage plays a critical role in supporting both access to PrEP medication as well as the associated clinical services. Our findings suggest that future efforts to expand PrEP access should continue to leverage Medicaid as a mechanism for reducing financial barriers to its use. Ensuring that such financial support remains in place is essential for sustaining and building upon the progress made in recent years.

The results of the study also brought attention to the problem of health disparities, even when an intervention is in place [20, 37]. We discovered that males, who saw a higher percentage increase in PrEP rate and PNR, benefited more from Medicaid expansion’s impact on outcomes than did women. Several studies have reported similar findings [18, 20, 35, 38]. These findings highlight the importance of examining data thoughtfully. Medicaid expansion is a macro-level intervention that is expected to benefit all recipients covered under the program. However, according to the study’s findings, men are far more likely than women to benefit from Medicaid coverage in terms of PrEP use. Nevertheless, while our study found greater improvements in PrEP uptake among men, even modest gains among women—particularly those from vulnerable or marginalized populations—are meaningful. These gains highlight the importance of investment in initiatives that reach women and address the unique challenges they face. Medicaid provides a foundation for such work. Its role in supporting the development and implementation of targeted interventions should be prioritized. Continued expansion of access, paired with tailored strategies that address the social and structural barriers women encounter, are vital to achieving equitable PrEP distribution and reducing disparities in HIV prevention.

There are numerous factors that could elucidate why women are less likely to benefit from PrEP care. First, it is important to acknowledge that women’s experience with sexually related healthcare is fundamentally different from that experienced by men. In general, women are faced with a myriad of sociostructural challenges that impact their interaction with the healthcare system and therefore serve as barriers to PrEP [39]. PrEP use has been highly stigmatized, with users being deemed as sexually irresponsible [40]. In most cultures, women face greater stigma surrounding their sexual health and HIV prevention preferences, which serves to discourage their motivations to use PrEP for fear of being judged [41, 42]. Black women are even further disadvantaged due to intersectional adversities, including racial discrimination, which hinder their access to PrEP [43]. In addition, many women’s agency and/or power to take charge of their own health and HIV prevention may be thwarted by power imbalances in relationships, financial dependence on partners, and intimate partner violence [42]. Other barriers to PrEP include transportation and childcare [44]. When women have competing demands, it is reasonable to conclude that they are less likely to be engaged in PrEP care that is delivered in ways that do not take that into consideration (e.g., standard clinic hours, in person visits) [45].

Women also have concerns of the medication’s side effects and impacts on reproductive health [43, 46, 47]. These are not unfounded since clinical trials with women have been limited [48, 49]. PrEP education, research and marketing have been historically targeted at men leaving women with less information about their options. As a consequence, most women who could benefit from the medication do not see themselves in how PrEP is marketed [40, 50]. Also, many providers lack understanding of the relevance and importance of PrEP for women, the ability to identify candidates who could benefit, and some struggle with initiating conversations surrounding its use [51–53]. Among women, there is also the issue of low risk perception since assessments more often focused on men, particularly men who have sex with men (MSM) [54–56].

Due to medical racism and historical harm in healthcare, women of color and those from marginalized groups may have mistrust of the healthcare system [46, 57]. Black women, in particular, are less likely to be introduced to PrEP by their healthcare providers [43]. Granted, men of color also face systemic mistrust in the healthcare system, and non-White men receive less benefit from PrEP than White men [58]. However, when added to the existing barriers women face in navigating a health system that is intimidating, medical mistrust exacerbates the issue of low PrEP access among Black women, which is the female demographic most impacted by HIV [57].

In order to better reach women placed at risk of HIV, policymakers should consider more targeted interventions that can complement large-scale initiatives, such as Medicaid expansion, thereby achieving health equity for women [37, 38]. New York has seen a significant increase in PrEP use among Medicaid enrollees due to statewide efforts to promote the medication [38]. Seiler et al. (2022) proposed recommendations for equitable distribution beyond cost barriers, including promoting PrEP through reimbursement and provider networks, closely monitoring existing programs, and expanding access through alternate avenues [59]. The authors acknowledged the need for more targeted approaches to offer PrEP to marginalized groups. PrEP interventions addressing women’s unique challenges and barriers are needed. Increasing PrEP usage and guaranteeing its equitable distribution requires more than just removing the cost barrier; additional steps are needed to make the drug more accessible to the women who need it most.

## Implications

While Medicaid expansion appears to increase access to PrEP overall, our findings suggest that additional efforts are needed to ensure these gains are equitably distributed. Cost is a significant barrier to PrEP access, and Medicaid plays a vital role in alleviating this obstacle by supporting both medication and related clinical care. This study also highlights gender disparities in PrEP use, with men being more likely to receive PrEP than women [11]. Efforts to improve PrEP uptake among women should build on this foundation by supporting tailored interventions that address barriers unique to women, particularly those from marginalized communities. Medicaid currently allows access to healthcare and PrEP services. Research could further explore the interaction of Medicaid and other targeted PrEP services and their ensuing effect on PrEP uptake and HIV outcomes in women. In addition, researchers should explore ways to optimize existing interventions to better serve women. For example, it would be beneficial to examine the factors that drive PrEP uptake in men so that they can be adapted to improve outcomes in women. Alternatively, further research should also explore factors that serve as barriers to PrEP uptake in women to help with determining non-traditional ways of identifying, engaging and sustaining women vulnerable for HIV in PrEP care.

Nevertheless, even small increases in PrEP uptake among women should be recognized as progress, given the longstanding challenges in reaching this population. Optimizing the use of Medicaid alongside tailored strategies has the potential to improve equitable access to PrEP and advance national goals to end the HIV epidemic. Any attempt to limit the funds and the scope of Medicaid could result in setbacks in accomplishing equitable PrEP use. Additional research is needed to identify effective strategies for identifying women with reasons to prevent HIV and determine which strategies are most cost-effective. This knowledge will be crucial in program planning and resource allocation for expanding PrEP reach. The findings would advance the goal of ending the HIV epidemic by 2030.

## Limitations

We acknowledge that there are some limitations to the study. First, some organizations do not release their data on PrEP prescriptions. Therefore, this might have resulted in an underestimation of PrEP use. In addition, PrEP use data disaggregated by race/ethnicity was not available for all states and years. Neither was the disaggregation by gender (instead of sex) available therefore preventing analyses and discussion of transgender women, men, and nonbinary individuals. Based on the type of analysis conducted for the study, we were not able to capture these differences using the incomplete data. Additionally, as with other PrEP/HIV surveillance datasets, the PrEP data was categorized to include both adults and adolescents. As a result, we were unable to examine how the factors affected PrEP results for adults alone. This could have had an impact on our findings as well. The data on PrEP use is based on the number of filled prescriptions per person in a one-year period, which does not guarantee continued use in subsequent years, causing uncertainty surrounding changes in PrEP use. This could have therefore affected the interpretation of the results.

The PNR compares PrEP users to new HIV diagnoses across states but may be biased as, compared to expansion states, nonexpansion states are primarily in the South—the region most affected by the HIV epidemic [2]. Our results for the PNR may have been biased since nonexpansion states could be representing those regions with higher HIV incidence. Future research should investigate how predictors affect the PNR regionally.

## Conclusions

The National Institutes of Health (NIH) plans to end the HIV epidemic by 2030, requiring PrEP uptake. However, the rates of PrEP use among women are low. This study analyzed the impact of Medicaid expansion on PrEP use in women, comparing outcomes for men and women. Results showed that Medicaid expansion increased PrEP use in women, but the effects were minor compared to men. The study suggests further research to scale up PrEP use among women and emphasizes the need for effective ways to identify women with reasons to prevent HIV and engage them in PrEP care. It also highlights the importance of preserving Medicaid expansions as a means of sustaining improvements in HIV prevention. An all-hands approach is needed to combat the HIV epidemic and achieving equity in PrEP use.
